# Safety of Chinese Herbal Medicine for Chronic Obstructive Pulmonary Disease

**DOI:** 10.1155/2015/380678

**Published:** 2015-03-26

**Authors:** Meaghan Coyle, Johannah Linda Shergis, Shaonan Liu, Lei Wu, Anthony Lin Zhang, Xinfeng Guo, Chuanjian Lu, Charlie Changli Xue

**Affiliations:** ^1^Traditional & Complementary Medicine Research Program, School of Health Sciences and Health Innovations Research Institute (HIRi), RMIT University, Bundoora, Melbourne, VIC 3083, Australia; ^2^Guangdong Provincial Hospital of Chinese Medicine, Guangzhou 510120, China; ^3^The Second Clinical College, Guangzhou University of Chinese Medicine, Guangzhou 510405, China; ^4^Guangdong Provincial Academy of Chinese Medical Sciences, Guangzhou 510120, China

## Abstract

Chinese herbal medicine (CHM) is increasingly used by patients with chronic obstructive pulmonary disease (COPD); however, there has been no systematic evaluation of its safety. This review examined the adverse events (AEs) reported in clinical studies of CHM for COPD. Five English databases (PubMed, Embase, CINAHL, AMED, and CENTRAL) and four Chinese databases (CBM, CNKI, CQVIP, and Wanfang Data) were searched from inception to May 2013. Adverse event data, including nature, severity, author-assigned causality, management, and outcome, were extracted from included studies. Descriptive statistics were used for the rate of adverse events. Of the 152 included studies, AEs were reported in 47 studies. The rate of adverse events was slightly lower in the CHM groups compared with controls (84 events in 5,909 participants, 1.4% versus 102 events in 5,676 participants, 1.8%). The most frequently reported adverse event was nausea (28 cases in the CHM groups and 19 cases in the control groups), which was more common in studies where CHM was combined with pharmacotherapy to treat acute exacerbation of COPD. Other frequent adverse events were abdominal discomfort, dry mouth, and dizziness. Detailed information about the adverse events was scant. Overall, CHM appears to be well tolerated in people with COPD.

## 1. Introduction

Chronic obstructive pulmonary disease (COPD) is a persistent disease with recurrent daily symptoms. Despite available drug treatments such as bronchodilators and steroids, 20–43% of people with COPD also use complementary therapies, including Chinese herbal medicine (CHM), to alleviate their symptoms [1, 2]. The frequent use of CHM by COPD patients may be underpinned by their belief that herbal medicines are safe [1, 3]. Patients taking complementary therapies felt that these therapies did not have risks because of their “naturalness” [1].

CHM appears to have clinically beneficial effects with very low risk of adverse events. Recent systematic reviews demonstrated that CHM improved lung function [4] and quality of life [5]. While many of the studies included in these reviews did not report adverse events (AEs), a number of studies included information concerning minor events. Therefore, these authors concluded that CHM was overall well tolerated by COPD patients [4, 5].

Without specific reference to COPD, one prospective study on general safety of a range of CHM interventions over a four-week period showed that 14% of patients reported an AE which was associated with CHM [6]. The most common AE was diarrhoea (4.2 events per 100 patients over 4 weeks). Hong Kong Hospital data revealed that, in an eight-month period, 0.2% of admissions were associated with AE from CHM [7]. The AEs were mostly minor; however, some were severe. Several reviews of AEs associated with CHMs have described the nature of the AE, but not the number of events [3, 8–10], often due to omissions in included studies. Finally, one study reported one adverse event per 633 consultations with a CM practitioner (for either CHM treatment or acupuncture) [11].

Due to the wide ranging CHM formulae used for COPD and the generic term “Chinese herbal medicine” not commonly used, combined with variability in publication keywords, it is difficult to identify relevant studies through electronic database searching. To date, there has been no specific evaluation of the safety of CHM for people with COPD based on clinical research evidence. Therefore, we systematically reviewed AEs reported in clinical studies of CHM for COPD. This review describes the incidence of AEs including nature, severity, and relationship with CHM treatment and was undertaken as part of a broader review of the evidence of efficacy (reported elsewhere).

## 2. Materials and Methods

The search strategy was guided by the Cochrane Airways Group methodology [12]. Nine databases were searched from inception to May 2013 (English: PubMed, Embase, CINAHL, AMED, and CENTRAL; Chinese: CBM, CNKI, CQVIP, and Wanfang Data). For the broader review that evaluated CHM efficacy for treating COPD, studies were included if they used CHM alone or combined with pharmacotherapy compared to pharmacotherapy, placebo, or no treatment. All forms of CHM were included, such as oral, intravenous, and intramuscular. Studies were eligible if they reported on adverse events in addition to any one of the following outcomes: lung function (FEV_1_, FVC), symptom severity (dyspnoea scales mMRC scale, Borg scale, and DVAS), exercise capacity (6 MWT), arterial blood gases (PaO_2_, PaCO_2_), COPD exacerbations, BODE index, or effective rate. Controlled clinical trials and noncontrolled studies published in English and Chinese were included. Studies which did not report the number of people randomised to the treatment and control groups (for controlled studies) were excluded.

The title and abstract of retrieved citations were screened for eligibility, and full text was retrieved where necessary. Eligibility assessment was confirmed by a second person. Study characteristics and AE data were extracted into a predefined form, with double-data extraction and consistency checking to ensure accuracy of the data. Studies which did not report on AEs were excluded from further analysis. Items relating to AEs included nature of AE and number of events, severity, and author-assigned causality, how the AE was managed, and the outcome. We used only the author-assigned causality (if reported) to avoid introducing bias, as we deemed it unlikely that sufficient information would be reported in the original publications to determine causality. For controlled studies, AEs were described according to group allocation or separately if group allocation was not specified by the study author. Where two events were reported together, for example, nausea and headache, these were considered as one incident for the purposes of this review.

The rate of AEs was calculated overall and according to CHM or control groups by dividing the number of events by the number of participants, expressed as a percentage. Similar AEs were grouped to allow for reporting of events by broad category. Analysis of time to event and multiple events per participant were planned but can not be performed due to insufficient details.

## 3. Results

**Figure 1:**
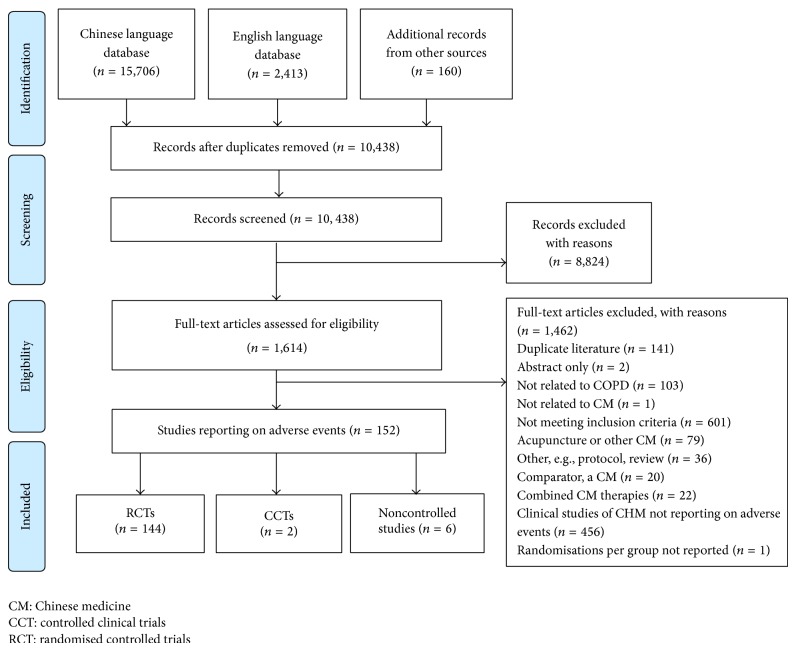
Flow diagram of included studies.

One hundred and fifty-two (152) of the 609 identified studies reported on AEs (occurrence or absence) (see Figure 1). The majority of studies were RCTs (144 studies), while two were nonrandomised controlled trials and six were noncontrolled studies. A total of 11,999 participants were included in this analysis, with 6,261 receiving treatment with CHM and 5,676 people allocated to control groups. The age of participants ranged from 18 to 92 years, and the median of mean age was 65.6 years. Duration of treatment ranged from three days to one year, with a median duration of two weeks. The most common CHM was* Tan re qing* injection (27 studies).

Adverse events occurred in 47 studies (involving 4,057 participants) [13–59] (see Table 1). A total of 207 events were reported (11,999 participants, 1.7%). In the controlled studies, 84 events occurred in those who received CHM intervention (5,909 participants, 1.4%) and 102 events in those who were allocated to the control groups (5,676, 1.8%). Nausea was the most frequently reported AE in both CHM and control groups (occurring in 28 cases and 19 cases, resp.). The vast majority of nausea AEs occurred in people with acute exacerbation of COPD where CHM was combined with pharmacotherapy. Other frequently reported AEs included abdominal discomfort (13 cases in CHM, 3 cases in control), dry mouth (7 cases in CHM, 9 cases in control), and dizziness (4 cases in CHM, 10 cases in control).

For analysis, studies were grouped according to intervention categories: (1) CHM alone or (2) CHM combined with pharmacotherapy and by study design: (1) controlled studies and (2) noncontrolled studies. Events were categorised into three broad groups: gastrointestinal events, dermatological events, and miscellaneous events (see Table 2). The CHM formulae and ingredients in studies where AEs occurred in the CHM groups are described in Table 3.

### 3.1. Controlled Studies

#### 3.1.1. CHM Alone

Eight studies compared CHM to controls and six reported the occurrence of AEs [39, 40, 44, 48, 50, 58]. Two studies reported that no events occurred [60, 61]. CHM was compared with placebo [44, 60], no treatment [58], or pharmacotherapy [39, 40, 48, 50, 61]. Twenty-nine (29) AEs were reported in the six studies. Thirteen of these AEs occurred in the treatment groups and 16 events in the control groups.

The number of gastrointestinal AEs was lower in those who received CHM compared with controls (2 AEs versus 6 AEs) but similar for miscellaneous AEs (11 AEs versus 10 AEs) (see Table 2). No dermatological AEs were reported in both the CHM and comparator groups. The most frequently reported event in the CHM groups was shivering/shaking hands (3 events), while bloating was the most frequent AE in the comparator groups (3 events). In studies which described the severity of AEs, all were reported as mild with the exception of three infections which were judged as severe and lead to patient death. One death occurred in the treatment group but was deemed unlikely to be related to treatment and two in the control group of the same study [58]. In one study that evaluated* Mai men dong tang* (*Bakumondoto*) two cases of elevated alkaline phosphatase (ALP) were considered possibly related to treatment; however, retest was not performed and a full exploration of causality could not be determined [39]. To manage AEs, study investigators changed the CHM dose [50], prescribed other drugs [48], continued treatment as allocated [48, 50], or did not administer treatment [40], or the patients withdrew [58].

#### 3.1.2. CHM Plus Pharmacotherapy

One hundred and thirty-eight (138) studies combined CHM with pharmacotherapy, and 37 of these reported the occurrence of 167 AEs [13–30, 32–34, 36–38, 41–43, 45–47, 49, 51–54, 56, 57]. In the intervention group, CHM was given with the same pharmacotherapy as the control group. Eighteen studies did not specify the pharmacotherapy used [13–16, 19, 20, 23, 25, 26, 29, 33, 34, 38, 47, 49, 51, 53, 54]. Antibiotics were used in 15 studies, methylxanthines in 14 studies, mucolytic agents in seven studies, bronchodilators and steroids in three studies each, and combined bronchodilator/inhaled corticosteroid in one study.

Fewer adverse events were reported in those who received CHM plus pharmacotherapy (71 events) compared with the pharmacotherapy alone (86 events). For a small number of AEs, the group allocation was not specified (10 events).

Adverse events were analysed according to the nature of the event (see Table 2). The number of gastrointestinal adverse events was greater in CHM plus pharmacotherapy group than pharmacotherapy alone (35 versus 26 events), while the reverse was found for miscellaneous AEs (31 versus 48, resp.). Nausea was the most frequently reported AE, occurring in 27 events in the CHM plus pharmacotherapy group and 18 events in the pharmacotherapy alone group. Few dermatological events were reported and were similar between groups (10 in CHM plus pharmacotherapy, seven in pharmacotherapy alone). Intravenous injection with Tan re qingcombined with pharmacotherapy was associated with all the dermatological events in the treatment group. A broad range of other AEs were seen. Other frequently reported AEs were dry mouth/throat (5 cases in the treatment groups and 9 cases in the control groups) and dizziness (3 cases in the treatment groups and 6 cases in the control groups).

In studies where the severity of the AE was reported, all events were described as mild. Sixteen AEs were described as being probably related to CHM plus pharmacotherapy interventions [18, 21, 24, 28, 33, 37, 38, 41, 43, 46, 52], five events were possibly related to treatment [29], and one was unlikely to be related to treatment [22]. Nine reports of AEs in the pharmacotherapy group were considered probably related to treatment [18, 28, 37, 38, 43, 46] and five possibly related to treatment [29]. Management of AEs was reported in most studies and subsequently all the events resolved. Management included dosage change for CHM [15, 33, 46], prescription of other drugs and/or hospital admission [15, 18, 20, 24, 26, 28, 41, 43, 52], and no treatment [20, 22], and in 11 studies the AE was able to be tolerated whilst continuing treatment [14, 16, 23, 25, 30, 36–38, 42, 54, 56].

### 3.2. Noncontrolled Studies

Six case series report on AEs [31, 35, 55, 59, 62, 63]. Adverse events occurred in four studies, two where CHM was used alone [55, 59] and two where CHM was combined with pharmacotherapy [31, 35]. Nine AEs were reported in those who received CHM alone, and two events occurred where CHM was combined with pharmacotherapy. Exacerbation of COPD was the most common AE in the noncontrolled studies (5 events). Exacerbations were managed with antibiotics [55]. Other AEs included vomiting (2 events) [59], and one case each included nausea [59], headache and dizziness [59], abdominal discomfort and nausea [35], and patient death (reason not given) [31]. None of the studies reported the severity of the AE or assigned causality. The one participant who reported abdominal discomfort and nausea continued treatment [35], and the headache and dizziness in one participant resolved without intervention [59].

## 4. Discussion

Overall, the rate of AEs in clinical studies of CHM for COPD was low (1.7%). The rate of AEs in participants who received CHM intervention was slightly lower than control groups, 1.4% versus 1.8%. This result fell well within the range of minimal risk for minor complications (0.1% to 10%) suggested by MacPherson et al. [64]. Where described, the majority of AEs were mild in severity. CHM appears to be well tolerated by patients with COPD.

Nausea was the most common AE in both the treatment groups and control groups but was higher amongst those who received CHM. The majority of nausea AEs occurred in people with an acute exacerbation of COPD. It is difficult to determine whether this is related to exacerbation, the CHMs, or the pharmacotherapy administered concurrently. Whilst the number of AEs which included nausea was higher in those who received CHM, this was within the range for minimal risk [64] and overall the data shows that CHM in combination with pharmacotherapy does not pose any additional risks for people with acute exacerbations of COPD. The data shows no clusters of particular events. A causal relationship to treatments was not evident and the AEs may reflect general fluctuations in the patients' conditions.

Many of the pharmacotherapies used in included studies have established safety profiles and known AEs. For example, bronchodilators can cause tremor, tachycardia, headaches, nausea, palpitations, hypokalaemia, and dry mouth, and corticosteroids can cause pneumonia, oral candidiasis, and skin bruising [65]. Among studies of CHM plus pharmacotherapy versus pharmacotherapy alone the number of AEs was lower amongst those who received CHM. In clinical practice, the choice of CHM can be influenced by the known safety profile of particular pharmacotherapies, with herbs added to address known side effects. The finding from this review suggests that CHM may reduce the number of AEs in people with COPD, although further research is needed to confirm this finding as the differences seen were small.

People with COPD often have comorbidities requiring polydrug therapy, which may increase the potential for herb-drug interactions. Indeed, there is significant potential for adverse drug reactions in elderly people with complex and chronic health conditions such as COPD and asthma [66]. Elderly people with COPD who are taking CHM in addition to prescribed medications should be monitored for the emergence of new signs or symptoms and should be reviewed regularly.

The data presented in this review is important because there are no existing reviews of clinical studies on the safety of CHM for COPD. While population and practitioner surveys may be more reflective of clinical practice, AEs reported in population surveys may differ from those in clinical studies because of known controllable risks associated with CHM. These include misidentification of herbal medicines, substitution, lack of standardisation, contamination, and adulteration, as well as some herbal ingredients not being listed on the product labels [67, 68]. In the clinical trial setting, the herbal medicine(s) is more likely to be controlled for these issues and any subsequent AEs can be better understood in the context of specific causality rather than poor manufacturing or handling.

The challenges of conducting systematic reviews of CHM have been described by White [69]. This review found a lower rate of AEs than that of MacPherson and Liu [6]. Few systematic reviews have evaluated the safety of CHM, and none have focused on patients with COPD. This may in part be due to the many and varied herbal formulae used in clinical trials and variation in publication keywords which limits electronic database searching. Further, the incidence of AEs is challenging to calculate as it requires adequate reporting of AEs in clinical studies (e.g., number and nature of events and authors definition of AE), information on the number of consultations, and whether the CHM was changed during the course of treatment. Additionally, some studies only provided limited and vague statements about AEs, such as “there were no AEs” or “CHM was safe.” Therefore the actual frequency of AEs may be underestimated.

Reporting of details of AEs in studies included in this review was limited largely to the nature and number of AEs, with scant details on severity, causality, management, and outcome. As AE data can be influenced by the manner in which it is collected [70], future clinical studies should describe the process for collection of safety data. Further, AE data in future clinical studies of CHM for COPD should be reported in accordance with the CONSORT extension for reporting of harms in randomised trials [71].

Finally, the diversity of CHM formulae and individual herbs used in included studies did not allow for greater exploration of AEs by individual formula (with the exception of Tan re qing injection) or by individual herb. In CHM formula with multiple herb ingredients, it is difficult to attribute AEs to one herb or the synergistic actions of all herb ingredients. Thus, the findings of this review are limited to describing the potential for an AE when any CHM formula is used. The selection of CHM formula for people with COPD should consider relevant available safety data and the concomitant medications being used by the patient.

## 5. Conclusion

Overall, CHM is well tolerated by patients with COPD. The rate of AEs is low, minor, and self-resolving and was slightly less than that of pharmacotherapy, placebo, and no treatment. The most common AE was nausea, which was seen when CHM was combined with pharmacotherapy for acute exacerbations of COPD. In addition, CHM appeared to be safe when taken in combination with pharmacotherapies. Future research should provide greater details relating to AEs in order to more accurately inform clinical practice.

## Figures and Tables

**Table 1:** Characteristics of studies reporting adverse events.

Study author, year	Study design	Stage of COPD	Comparison	Sample size	Number randomised to treatment group/control group	CHM intervention; administration	Cointervention/comparator
Che HZ, 2005	RCT	Stable	2	50	25/25	Zhi ke qing fei liquid, oral	Pharmacotherapy
Chen R, 2005	RCT	AECOPD	2	66	36/30	Jin shui bao capsule, oral	Pharmacotherapy
Cui FS, 2013	RCT	AECOPD	2	69	35/34	Ma xing shi gan tang, oral	Multipharmacotherapies
Dong GB, 2008	RCT	AECOPD	2	240	120/120	Xi xin nao, IV	Pharmacotherapy
Fu ZY, 2008	RCT	Stable	2	60	30/30	Chen xia liu jun zi tang + San zi yang qin tang (modified), oral	Methylxanthines
Gong GL, 2008	RCT	AECOPD	2	96	53/43	Tan re qing, IV	Antibiotics plus mucolytics
Guo LX, 2005	RCT	AECOPD	2	60	30/30	Tan re qing, IV	Antibiotics plus methylxanthines plus mucolytic
Guo YR, 2011	RCT	AECOPD	2	60	30/30	Huang qi tang, oral	Multipharmacotherapies
Han GL, 2011	RCT	AECOPD	2	68	37/31	Hua zhuo qu yu tang, oral	Pharmacotherapy
Hu Y, 2010	RCT	AECOPD	2	94	34/28	Tan re qing, inhaled	Antibiotics plus methylxanthines plus mucolytic
Hua ZQ, 2013	RCT	AECOPD	2	98	50/48	Yi qi qing fei tang, oral	Pharmacotherapy
Huang B, 2006	RCT	AECOPD	2	60	30/30	Tan re qing, IV	Multipharmacotherapies
Huang DH, 2005	RCT	Stable	2	63	32/31	Yu ping feng ke li + Bai ling capsule + Jian pi yi fei chong ji, oral	Pharmacotherapy
Ji, 2003	CCT	Stable	2	19	10/9	CHM formula (unnamed), oral	Pharmacotherapy
Li L, 2011	RCT	Stable	2	70	35/35	Shen ge jiao nang, oral	Methylxanthines plus placebo
Li DL, 2009	RCT	AECOPD	2	60	30/30	Xuan bai chen qi tang, oral	Antibiotics plus methylxanthines plus mucolytic
Li FY, 2009	RCT	AECOPD	2	96	64/32	Tan re qing, IV	Antibiotics plus mucolytics
Li PW, 2010	CS	NS	4	68	NA	Wen qing di tan yin (modified), oral	Pharmacotherapy
Li SY, 2012	RCT	Stable	2	352	176/176	Bu fei jian pi or Bu fei yi shen or Yi qi zi shen, oral	Pharmacotherapy
Liang AL, 2009	RCT	Stable	2	60	30/30	CHM formula (unnamed), oral	Salmeterol/fluticasone and methylxanthines
Liu JB, 2012	RCT	AECOPD	2	62	31/31	Tan re qing, IV	Pharmacotherapy
Liu W, 2011	RCT	AECOPD	2	62	32/30	Xie fei tang + Li pi bu shen tang (modified), oral	Pharmacotherapy
Liu XJ, 2005	CS	AECOPD	4	126	NA	Tan re qing, IV	Pharmacotherapy
Liu YQ, 2006	RCT	AECOPD	2	83	43/40	Jin shui bao jiao nang, oral	Antibiotics plus methylxanthines plus steroids
Ma XC, 2009	RCT	AECOPD	2	112	60/52	Tan re qing, IV	Multipharmacotherapies
Ming ZQ, 2008	RCT	AECOPD	2	40	20/20	Xue bi jing, IV	Pharmacotherapy
Mukaida, 2011	RCT	Stable	1	24	13/11	Mai men dong tang (Bakumondoto), oral	Pharmacotherapy
Ou L, 2012	CCT	AECOPD	1	82	41/41	Jin kui shen qi pill + Ma xing shi gan formula + Xiao qing long formula + Bu fei formula, oral	Multipharmacotherapies
Peng WG, 2008	RCT	AECOPD	2	87	44/43	Tan re qing, IV	Antibiotics plus methylxanthines plus mucolytic
Shen YD, 2010	RCT	Stable	2	81	32/49	Gu ben ke chuan pill, oral	Ipratropium bromide plus routine care
Tong WN, 2012	RCT	Stable	2	82	42/40	Xiao ke chuan jiao nang, oral	Bronchodilator
Wu, 2011	RCT	Stable	1	263	178/85	Jian pi yi fei II (modified), oral	Placebo
Xiong CW, 2011	RCT	AECOPD	2	112	56/56	Fu zheng hua tan qu yu tang (modified), oral	Pharmacotherapy
Yang XJ, 2006	RCT	AECOPD	2	50	25/25	Tan re qing, IV	Antibiotics plus bronchodilator plus mucolytic
Ye, 2010	RCT	Stable	1	90	45/45	Bu yuan tang, oral	Pharmacotherapy
You BW, 2007	RCT	Stable	2	64	32/32	Yi fei he ji, oral	Methylxanthines plus a bronchodilator plus steroid
Yuan YL, 2011	RCT	AECOPD	2	80	40/40	Sang bai pi tang (modified), oral	Pharmacotherapy
Zhai, 2012	RCT	Stable	1	140	70/70	Fu zheng qing tan hua yu, oral	Drug therapy
Zhang, 2008	RCT	Stable	1	73	38/35	Liu jun zi pill, oral	No treatment
Zhang LH, 2011	RCT	AECOPD	2	80	40/40	Su zi jiang qi tang, oral	Antibiotics plus methylxanthines plus mucolytic
Zhang Y, 2004	RCT	AECOPD	2	60	30/30	Tan re qing, IV	Antibiotics plus methylxanthines plus mucolytic
Zhang YF, 2010	CS	NS	3	45	NA	Yang he tang (modified), oral	NA
Zhao W, 2012	RCT	AECOPD	2	62	31/31	CHM formula (unnamed), oral	Multidrug therapy
Zheng H, 2012	RCT	AECOPD	2	60	30/30	CHM formula (unnamed), oral	Pharmacotherapy
Zheng XG, 2003	CS	Stable	3	60	NA	Yi xin kang tai jiao nang, oral	NA
Zhong HW, 2010	RCT	Stable	2	87	44/43	Fei shu jiao nang, oral	Methylxanthines alone
Zhou J, 2008	RCT	AECOPD	2	81	43/38	Tan re qing, IV	Antibiotics plus methylxanthines plus mucolytic

AECOPD: acute exacerbation of COPD; CCT: controlled clinical trial; CHM: Chinese herbal medicine; CS: case series; I: intervention; IV: intravenous; NA: not applicable; NS: not specified; RCT: randomised controlled trial.

Comparison column: 1: CHM versus control; 2: CHM plus pharmacotherapy versus pharmacotherapy; 3: CHM (case series); 4: CHM plus pharmacotherapy (case series).

**Table 2:** Summary of adverse events.

Intervention type	Group	Gastrointestinal events	Dermatological events	Miscellaneous events
Controlled trials				
CHM (*n* = 6) [39, 40, 44, 48, 50, 58]	Treatment	**Total events: 2** Diarrhoea: 2	**None**	**Total events: 11** Not specified: 5 Shivering/shaking hands: 3 Elevated alkaline phosphatase (ALP): 2 Infection: 1
	Control	**Total: 6** Nausea/bloating: 3 Bloating: 3	**None**	**Total: 10** Not specified: 3 Infection: 2 Insomnia: 1 Limb tremor: 1 Tachycardia: 1 Cerebrovascular accident: 1 Heart failure: 1

CHM + pharmacotherapy (*n* = 38) [13–30, 32–34, 36–38, 41–43, 45–47, 49, 51–54, 56, 57]	Treatment	**Total: 33** Nausea and abdominal distension: 9 Nausea: 8 Nausea and vomiting: 6 Gastrointestinal reaction (unspecified): 4 Nausea and diarrhoea: 2 Nausea and stomach upset: 2 Constipation: 1 Abdominal distension: 1	**Total: 10** Rash/red papules: 7 Itching: 3	**Total: 28** Dry mouth: 5 Exacerbation of COPD: 5 Dizziness: 3 Upper respiratory tract infection: 2 Dry mouth and abdominal distension: 2 Acid regurgitation and chest distress: 2 Nose injury (trauma due to breathing mask): 2 Headache: 1 Hypertension: 1 Insomnia: 1 Irritability: 1 Palpitation: 1 Pulmonary encephalopathy: 1 Thirst: 1
	Control	**Total: 26** Nausea: 8 Nausea and vomiting: 4 Nausea and dizziness: 4 Diarrhoea: 4 Nausea and abdominal distension: 2 Abdominal distension: 1 Loss of appetite: 1 Constipation: 1 Stomach discomfort: 1	**Total: 7** Rash: 6 Blister: 1	**Total: 53** Dry mouth/throat: 9 Dizziness: 6 Upper respiratory tract infection: 6 Nose injury (trauma due to breathing mask): 4 Palpitation: 4 Pulmonary encephalopathy: 3 Irritability: 3 Fungal infection: 3 Exacerbation of COPD: 2 Hypertension: 2 Leukocyte <4 × 10^9^/L: 2 Lung infection: 2 Insomnia: 1 Black stool: 1 Allergy to antibiotics: 1 Platelet <100 × 10^9^/L: 1 Sinus tachycardia: 1 Thirst: 1 Not specified: 1
	Group not specified	**Total: 10** Abdominal distension: 5 Gastrointestinal reaction (unspecified): 3 Diarrhoea: 2	**None**	**None**

Case series				
CHM (*n* = 2) [55, 59]	NA	**Total: 3** Vomiting: 2 Nausea: 1	**None**	**Total: 6** Exacerbation of COPD: 5 Headache and dizziness: 1

CHM + pharmacotherapy (*n* = 2) [31, 35]	NA	**Total: 1** Abdominal discomfort and nausea: 1	**None**	**Total: 1** Death (cause unknown): 1

**Table 3:** CHM formula and ingredients of studies with adverse events in treatment groups.

CHM intervention; route of administration	Herbal ingredients	References
Bu fei jian pi or Bu fei yi shen or Yi qi zi shen, oral	Bu fei jian pi: huang qi, dang shen, bai zhu, fu ling, and chuan bei mu; Bu fei yi shen: ren shen, huang qi, gou qi zi, shan zhu yu, and yin yang huo; Yi qi zi shen: ren shen, huang jing, shu di huang, mai dong, and wu wei zi	[29]

Chen xia liu jun zi tang + San zi yang qin tang (modified), oral	Dang shen, bai zhu, fu ling, chen pi, fa xia, su zi, lai fu zi, bai jie zi, tao ren, zhe bei mu, bei xing, hai ge qiao, and gan cao	[17]

CHM formula (unnamed), oral	Ma huang, bai guo, sang bai pi, zhe bei mu, gua lou, huang qin, su zi, zi he che, and ge jie	[54]

CHM formula (unnamed), oral	Fa ban xia, fu ling, chuan xiong, chao gu ya, chao mai ya, bai he, shan yao, sha shen, fo shou, shan zha, jin fei cao, and zhe bei mu	[26]

Fei shu jiao nang, oral	Hong shen, ge jie, san qi, chuan bei mu, di long, chuan xiong, and gan cao	[56]

Fu zheng hua tan qu yu tang (modified), oral	Zhi fu zi, bu gu zi, ting li zi, chuan xiong, and yu jin	[45]

Fu zheng qing tan hua yu, oral	Sheng shai shen/ren shen, ha jie, wu wei zi, xing ren, gua lou, xie bai, dan shen, tao ren, and mao dong qing	[50]

Gu ben ke chuan pill, oral	NS	[42]

Hua zhuo qu yu tang, oral	Chi shao, dan shen, tao ren, hong hua, gua lou, su mu, zi su zi, zhe bei mu, fu ling, kuan dong hua, yu xing cao, and gan cao	[21]

Huang qi tang, oral	Huang qi, dang shen, bai zhu, gua lou, xing ren, tao ren, chuan xiong, dang gui, chen pi, and huang qin	[20]

Jian pi yi fei II (modified), oral	Dang shen, bai zhu, suo yang, and tao ren	[44]

Jin kui shen qi pill + Ma xing shi gan formula + Xiao qing long formula + Bu fei formula, oral	Jin kui shen qi pill: shu di huang, shan zhu yu, shan yao, shu fu zi, gui zhi, bu gu zhi, fu ling, tao ren, ze xie, and wu wei zi Ma xing shi gan formula: ma huang, xing ren, huang qin, jie geng, sheng shi gao, yu xing cao, jin yin hua, lian qiao, zhe bei, tao ren, and gan cao Xiao qing long formula: gui zhi, bai shao, gan jiang, ma huang, ban xia, zhe bei, xi xin, chuan xiong, wu wei zi, and gan cao Bu fei formula: shu di, huang qi, dang shen, sang bai pi, zi yuan, kuan dong hua, qian hu, chen pi, wu wei zi, dan shen, su zi, and bai jie zi	[40]

Jin shui bao jiao nang, oral	Jin shui bao jiao nang	[14, 36]

Liu jun zi pill, oral	NS	[58]

Ma xing shi gan tang, oral	Ma huang, xing ren, shi gao, gan cao, lai fu zi, ting li zi, fu ling, jv hong, zhu ru, shi chang pu, yu jin, gui zhi, fu ling, bai zhu, zhu ling, and ze xie	[15]

Mai men dong tang (Bakumondoto), oral	Mai men dong, ban xia, gu ya, da zao, ren shen, and gan cao	[39]

Shen ge jiao nang, oral	Ren shen, chuan bei, zi he che, and zhi ge jie	[30]

Tan re qing, inhaled	NS	[22]

Tan re qing, IV	Huang qin, jin yin hua, lian qiao, xiong dan fen, and shan yang jiao	[18, 19, 24, 28, 33, 35, 37, 41, 46, 52]

Wen qing di tan yin (modified), oral	Jiang ban xia, bei xi xin, wu wei zi, gan jiang, ting li zi, fu ling, and wang jiang nan. If yin deficiency, add sha shen, mai dong; if phlegm heat, add sang bai pi, gua lou pi	[31]

Xi xin nao, IV	NS	[16]

Xiao ke chuan jiao nang, oral	Man shan hong	[43]

Xue bi jing, IV	NS	[38]

Yang he tang (modified), oral	Su di, su zi, rou gui, gan cao, zhi ma huang, lu jiao jiao, bai jie zi, fa xia, shu mu, chen pi, and pao jian. If with cough, add chuan bei, zhi zhi wan; if with phlegm, add gua lou ren, cao miao zi; if spleen qi deficiency, add dang shen, bai zhu; if blood stasis, add dan shen, tao ren, yi mu cao; if with heat, remove rou gui, lu jiao jiao, and su di and add sang bai pi, zhe bei, se gan, and huang qin	[59]

Yi qi qing fei tang, oral	Huang qi, dan shen, huang qin, bei mu, ban xia, jie geng, gua lou qiao, and yu xing cao	[23]

Yi xin kang tai jiao nang, oral	Tang gu te qian xian lian, duo xian xian gou zi, tang gu te da huang, and shuo yang	[55]

Yu ping feng ke li + Bai ling capsule + Jian pi yi fei chong ji, oral	Yu ping feng ke li: NS; Bai ling capsules: NS; Jian pi yi fei chong ji: ren shen, bai zhu, fu ling, mai dong, sang bai pi, and huang qi	[25]

IV: intravenous; NS: not specified.
